# Methylphenidate in Borderline Personality Disorder: Assessing Its Therapeutic Potential and Limitations

**DOI:** 10.3390/life15030380

**Published:** 2025-02-27

**Authors:** Simone Pardossi, Alessandro Cuomo, Despoina Koukouna, Mario Pinzi, Bernardo Firenzuoli, Andrea Fagiolini

**Affiliations:** Department of Molecular Medicine, School of Medicine, University of Siena, 53100 Siena, Italy; alessandro.cuomo@unisi.it (A.C.); koukounadespoina@gmail.com (D.K.); mario.pinzi@student.unisi.it (M.P.); bernardo.firenzuoli@gmail.com (B.F.); andrea.fagiolini@unisi.it (A.F.)

**Keywords:** methylphenidate, impulsivity, borderline personality disorder

## Abstract

Impulsivity is increasingly recognized as a transdiagnostic feature that spans multiple psychiatric disorders, including borderline personality disorder (BPD), bipolar disorder, and substance use disorders. In BPD, impulsive behaviors manifest as substance misuse, risky sexual activity, self-injury, and other maladaptive patterns. This review article updates the clinical and preclinical literature to explore the biological and psychological bases of impulsivity in BPD and considers whether methylphenidate (MPH) can be used as a treatment in this context. Although no medication is specifically approved for BPD, limited evidence from patients with comorbid BPD and attention-deficit/hyperactivity disorder (ADHD) indicates that MPH may reduce impulsivity and improve key symptoms. In addition, real-world data indicate that MPH may be associated with better outcomes and a lower risk of suicidal behaviors in patients with BPD. Nevertheless, such evidence remains scant, particularly among those with a primary diagnosis of BPD without a diagnosis of ADHD. Larger, methodologically rigorous studies are needed to clarify the efficacy and safety of MPH in targeting impulsivity within this population. An improved understanding of dopaminergic mechanisms may eventually shed light on MPH’s therapeutic role in BPD, although current data remain preliminary. Overall, recognizing impulsivity as a core symptom rather than focusing exclusively on diagnostic boundaries may facilitate more tailored and effective interventions for BPD.

## 1. Introduction

Impulsivity is a complex and dynamic domain that shapes human decision-making and behavior, sometimes even characterizing it [1,2]. It is characterized by the propensity to act quickly and without forethought in response to internal and external stimuli with little or no consideration of the possible repercussions—both positive and negative—of doing so [1,2]. Such a propensity naturally has a tendency to lead to risky or maladaptive behavior that ignores long-term repercussions [1,2]. Within an exploration of the different elements that make up impulsivity, the central features involve problems with delaying gratification, managing immediate response, and staying committed to long-term objectives [3]. Although classically associated with conditions such as attention-deficit/hyperactivity disorder (ADHD) and substance use disorders (SUDs), impulsivity is now also understood as a transdiagnostic feature that cuts across several psychiatric conditions [4].

The neurobiological underpinnings of impulsivity entail intricate interactions among various brain areas that govern executive functions, emotional regulation, and reward processing [5]. The prefrontal cortex, more particularly the orbitofrontal cortex (OFC), is integral to decision-making and inhibitory control processes [6]. Structural and functional abnormalities in the OFC are consistently observed in disorders characterized by impulsivity, such as BPD [7]. Additionally, the ventral striatum is activated in the prediction of reward, while the amygdala signals emotional responses, exiting both circuits into impulsivity [8,9].

Neuroimaging research has demonstrated that impulsivity is related to decreased anterior cingulate cortex gray matter volume and dopaminergic circuit abnormalities between prefrontal and subcortical areas [10,11,12,13]. This type of evidence indicates that impulsivity may be the consequence of disrupted top-down inhibitory control with increased sensitivity to bottom-up reward processes, both of which are shared across psychiatric disorders [12].

Behaviorally, impulsivity can be conceptualized in a number of dimensions, including motor impulsivity (acting on impulse), attentional impulsivity (difficulty in staying focused), and cognitive impulsivity (making poor decisions) [14]. These are assessed using tools such as the Barratt Impulsiveness Scale (BIS) for self-reported impulsivity and behavioral tasks such as the Stop-Signal Task (SST) for measuring motor inhibition [15,16]. The BIS-11 is a self-report instrument measuring three impulsivity facets, namely, motor (acting without thinking), attentional (difficulty focusing), and non-planning (lack of future-oriented thinking) [15]. While no universal cutoff exists, higher scores indicate greater impulsivity [17]. The SST assesses the inhibition of a response, with a lower stop-signal reaction time indicative of impaired impulse control [18]. These scores are also useful for measuring poor impulse control, which is defined as the inability to resist urges or inhibit inappropriate behaviors, often leading to negative social, legal, or financial consequences [19].

In contrast to self-report measures that rely on an individual’s memory of what they have done, behavioral tasks provide empirical evidence of impulsivity in action. However, the heterogeneity of the expression of impulsivity across disorders renders it challenging to both assess and interpret. For example, patients with ADHD primarily exhibit motor impulsivity, whereas patients with substance use disorders exhibit decisional impairment and heightened reward sensitivity [20].

In spite of its fundamental contribution to research in psychopathology, impulsivity tends to be overlooked within clinical practice, largely as a result of the dominance of disorder-specific diagnostic frameworks like the DSM-5 [21]. Such traditional frameworks emphasize distinctive disorders, thus indirectly failing to account for transdiagnostic processes like impulsivity that traverse diagnostic categories. To rectify this deficit, emerging frameworks like the Research Domain Criteria (RDoC) and the Hierarchical Taxonomy of Psychopathology (HiTOP) encourage dimensional approaches that unite common neurobiological and behavioral characteristics [22,23].

Impulsivity is a hallmark of numerous psychiatric conditions, including BPD, bipolar disorder (BD), and SUDs, occurring in various forms that severely compromise both functional capacities and general quality of life. In BPD, impulsivity is associated with risky behaviors, such as drug abuse, self-mutilation, and suicidality, which have other widespread personal and social consequences [24,25]. The impulsivity trait has a number of dimensions, such as the motor dimension (acting without reflective consideration), the cognitive dimension (poor decision-making), and the emotional dimension (difficulty controlling affect) [24,25]. These tendencies are often triggered by intense emotion and have wide-ranging personal and social consequences [24,25]. Impulsivity is especially intense in the course of manic episodes of BD, in which it occurs in the form of impulsive, unplanned behaviors with reduced regard for possible consequences. This heightened impulsivity thereby increases hospitalization, suicidality, and the severity of the illness [26]. However, studies show that impulsivity can persist during depressive and euthymic stages, which indicates that it can be a trait-like feature of the illness [26]. Impulsivity plays a role in the development and maintenance of drug use in SUDs, most commonly through reduced cognitive control and reward sensitivity [27]. It is characterized by a lack of premeditation, lack of perseverance, sensation seeking, and urgency, all of which are accountable for substance-seeking behavior [27]. Aside from these indications, impulsivity is also closely associated with eating disorders, specifically in the instance of binge eating disorder, where it contributes to periods of reduced control over eating. Furthermore, in the instance of behavioral addictions, like pathological gambling, impulsivity enhances detrimental behavioral patterns [28].

In BPD, several psychotherapeutic approaches, i.e., schema therapy, dialectical behavior therapy (DBT), Systems Training for Emotional Predictability and Problem Solving (STEPPS), and mentalization-based therapy (MBT), have been shown to be effective in reducing impulsive behavior and enhancing emotional control [29]. While there is no pharmacological treatment specifically approved for BPD as of yet, it is still common practice to utilize antipsychotics, mood stabilizers, and antidepressants in patients with BPD [30]. In BD, mood stabilizers like lithium and anticonvulsants are pillars of pharmacologic therapy, together with antipsychotics [31]. Cognitive–behavioral therapy (CBT) is a mainstay in the management of SUD, emphasizing the recognition and alteration of adverse substance use-related thinking and behavior [32]. Contingency management (CM) is an approach that offers concrete rewards for behaviors that are wanted, such as abstinence from substances, and has been found to be effective in enhancing behavioral change in individuals with SUDs [33]. Pharmacological therapies for SUDs differ by substance [34]. Nevertheless, there is still not enough strong evidence for the pharmacological treatment of impulsivity.

Highlighting this specific field and domain can have significant relevance considering its transdiagnostic nature and its participation in disorders which are common as well as outcome-based. Furthermore, pharmacotherapies that use psychostimulants, like methylphenidate (MPH), are widespread in treating ADHD [35]. MPH has proven to be effective in improving the different facets of ADHD with an emphasis placed especially on impulsivity [36]. This efficacy has raised interest in investigating MPH for the treatment of other disorders, namely, those where impulsivity is a core characteristic, including BPD.

## 2. Methods

This narrative review aims to explore impulsivity as a transdiagnostic characteristic, with a particular focus on borderline personality disorder. To achieve this, we conducted a comprehensive literature search using databases such as PubMed, Scopus, and Web of Science, with keywords including “impulsivity”, “Borderline Personality Disorder”, “methylphenidate”, “ADHD”, “dopamine”, and “executive function”. Preclinical trials were included in order to provide a more thorough synthesis of evidence concerning MPH, its mechanisms of action, and effects on impulsivity. Furthermore, a thorough search was carried out in the mentioned databases for clinical studies on the nature of impulsivity in subjects with BPD with research on MPH as a possible treatment for these impulsivities. This complements the experimental data with real-world data to present a more balanced overview of the subject. Thus, this review examines the neurobiological underpinnings and clinical expression of impulsivity in borderline personality disorder, its comorbidity with attention-deficit/hyperactivity disorder, and the therapeutic potential of MPH for managing this core symptom. By synthesizing current evidence, we highlight the need for targeted treatment strategies and further research to refine interventions for impulsivity in borderline personality disorder, both in patients with and without comorbid attention-deficit/hyperactivity disorder.

## 3. Mechanism of Action of Methylphenidate: Targeting Impulsivity

MPH, a synthetic derivative of phenethylamine, is widely recognized for its efficacy in treating ADHD and its potential utility in comorbid conditions such as SUDs. MPH acts primarily by inhibiting the dopamine (DAT) and norepinephrine (NET) transporters, thereby increasing extracellular levels of dopamine (DA) and norepinephrine (NE) in the synaptic cleft. These effects are most prominent in the prefrontal cortex, a region associated with executive functions such as attention, working memory, and inhibitory control [37,38,39].

Beyond its primary mechanism of action, MPH has additional effects that contribute to its therapeutic profile. It interacts with adrenergic receptors, particularly activating α2 adrenergic receptors, which facilitates cortical excitability and further supports its procognitive effects. This adrenergic activity is evidenced by the ability of α2 receptor antagonists to block MPH’s effects in working memory tasks [40]. MPH also exhibits weak agonism at serotonin 1A (5-HT1A) receptors, indirectly enhancing dopaminergic signaling and contributing to neuroprotection [41]. This neuroprotective action involves regulating vesicular monoamine transporter 2 (VMAT2), reducing cytoplasmic dopamine accumulation and preventing the formation of neurotoxic reactive oxygen species, a mechanism particularly relevant in conditions like methamphetamine abuse and neurodegenerative diseases such as Parkinson’s [38]. MPH acts differently from other medications for ADHD, such as atomoxetine and guanfacine. The former is a selective norepinephrine reuptake inhibitor [42], while the latter is an alpha-2 adrenergic receptor agonist that modulates noradrenergic tone in the prefrontal cortex [43].

Neuroimaging studies have provided robust evidence of MPH’s effects on brain activity and functional connectivity. PET studies demonstrate MPH-induced increases in striatal dopamine availability, as reflected by reductions in radioligand binding to DAT, which correlate with changes in euphoria, anxiety, and task performance [39]. Functional MRI (fMRI) studies show that MPH enhances activation in the prefrontal and parietal cortices while deactivating regions such as the insula and posterior cingulate cortex during tasks requiring visual attention and working memory [44]. Moreover, MPH modifies the connectivity between the cortex and subcortex, prioritizing cortical activation among several regions such as the parahippocampal and cerebellar areas during uncertainty, and altering activity patterns in the anterior cingulate cortex and thalamus during inhibitory control and cognitive tasks [39].

Based on the already established effects of MPH on various neurochemical systems, the effects of MPH on impulsivity have previously been characterized in rodent models [45,46,47,48]. Research indicates that MPH administration can modulate impulsive behaviors, with outcomes varying based on factors such as dosage and the age and genetic background of the subjects [45,46,47,48]. MPH reduces impulsivity in animal models, especially in juvenile animals, producing improved delay tolerance and cognitive control [46]. However, the same dose is effective in young animals but is less effective in adult rodents, suggesting that age and neurodevelopmental factors are key modulators of its effects [46]. This variability emphasizes the role that genetic and neurobiological differences play in MPH’s effects on impulsivity [45,46]. Behavioral paradigms used to assess impulsivity, such as the 5-choice serial reaction time task (5-CSRTT) and T-maze delay discounting tasks, also reveal dose-dependent effects. Lower doses of MPH (e.g., 0.5–1 mg/kg) tend to reduce premature responses and improve inhibitory control, while higher doses may exacerbate impulsive actions, highlighting a narrow therapeutic window [47]. Additionally, neuroimaging studies in rodents indicate that MPH modulates activity in the prefrontal cortex and enhances functional connectivity in reward-related circuits during tasks requiring impulse control [47]. Finally, preclinical research on prenatal and early-life manipulations, such as alcohol exposure, suggests that MPH has limited efficacy in reversing impulsivity induced by early dopaminergic and noradrenergic dysfunction [48]. For example, in preadolescent rats prenatally exposed to alcohol, MPH only mildly reduces impulsivity, reinforcing the importance of developmental timing in shaping its therapeutic effects [48].

In human studies, MPH demonstrates robust efficacy in decreasing impulsive errors and enhancing self-control across a variety of tasks, including delay-of-reinforcement paradigms. For instance, acute MPH administration significantly increased the preference for larger, delayed rewards in adults, highlighting its role in promoting self-regulatory behavior [36]. Dougherty et al. expanded on this by demonstrating that MPH improves response inhibition in adolescents with ADHD and conduct disorder through tasks such as the Immediate Memory Task and Go/Stop Paradigm, which specifically target impulsive action and inhibition [49]. These findings indicate MPH’s potential to address core deficits in impulse control across a range of neuropsychiatric conditions. Neuroimaging studies further support this mechanism, as evidenced by the increased functional connectivity in executive control networks following MPH administration, implicating the prefrontal cortex as a key region underlying enhanced self-regulation [50]. Yet, patient heterogeneity highlights the contribution of baseline impulsivity, genetic antecedents, and comorbidities on MPH treatment response [39].

There is a growing body of research emphasizing the role of MPH in specific impulse subcomponents. For example, MPH has also been found to improve cognitive flexibility and reward processing, both essential functions for adapting to the changing demands of the environment and pursuing long-term goals [51].

It is noteworthy that, as mentioned before, MPH exerts its effects primarily by affecting the dopamine system. However, the relationship between dopamine levels and cognitive control (which is fundamental for reducing impulsivity) may follow an inverted U-shaped pattern: both excessively low and high dopamine levels can impair impulse control [52]. In this regard, selecting the appropriate MPH dosage is clinically relevant [53].

## 4. Impulsivity in Borderline Personality Disorder

### 4.1. Characteristics of Impulsivity in BPD

Impulsivity is a core feature of BPD and is also one of the diagnostic criteria outlined in the DSM-5-TR [3]. In the four-dimensional model proposed by Lieb et al., impulsivity is included as one of the principal components, alongside emotional instability, cognitive disturbances (such as episodes of dissociation or transient psychotic experiences), and instability in identity and interpersonal relationships [54].

**Figure 1:**
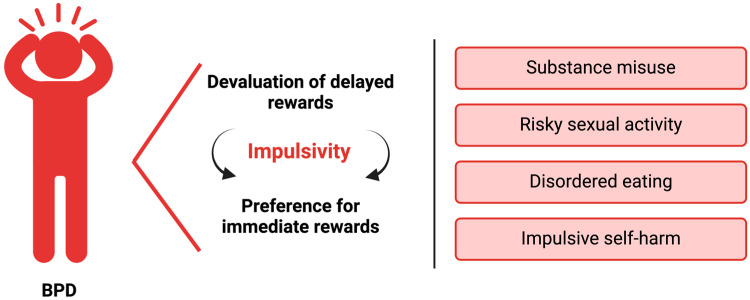
Impulsivity in borderline personality disorder (BPD).

Impulsivity in BPD manifests through behaviors such as substance misuse, risky sexual activity, disordered eating, and impulsive self-harm (Figure 1) [25,55,56]. Reward-based decision-making in BPD often favors immediate gratification, with a tendency to undervalue delayed rewards [57]. For instance, even with a short 24 h delay, individuals with BPD frequently opted for smaller immediate monetary rewards compared to controls, who demonstrated greater tolerance for delayed gratification [58]. Additionally, participants with BPD exhibited steeper delay discounting associated with higher general impulsivity and choice impulsivity, as measured by the BIS, although attentional and motor impulsivity did not correlate significantly [25,58].

One study assessed impulsivity in individuals with BPD by comparing 20 patients with BPD to 21 healthy controls using delay and probabilistic discounting tasks, as well as the SST [24]. The findings indicated that individuals with BPD exhibited significantly higher choice impulsivity, as evidenced by a preference for immediate rewards over larger delayed ones, and a greater tendency to devalue rewards based on their probability. Interestingly, no significant differences were observed in motor impulsivity between the two groups. These results suggest that choice impulsivity is a prominent feature in BPD, while motor impulsivity may not be as affected [24].

Impulsivity in BPD has been identified as a significant predictor of long-term outcomes. For instance, it was found to strongly predict borderline psychopathology at a 7-year follow-up [59]. Additionally, impulsivity has been linked to an increased risk of suicidal behavior in patients with BPD, with higher levels of impulsivity associated with a greater likelihood of suicide attempts [59,60,61].

Early manifestations of impulsivity may contribute to the development of maladaptive behaviors and heightened emotional sensitivity in patients with BPD. It has been suggested that impulsivity in childhood could lead to difficulties in emotion regulation and interpersonal relationships later in life, which are characteristic features of BPD [62].

Impulsivity is a core feature of BPD. In particular, it manifests through both a diminished valuation of delayed rewards and a preference for immediate rewards. These tendencies, in turn, contribute to various hallmark features of BPD, including substance misuse, risky sexual activity, disordered eating, and impulsive self-harm.

### 4.2. Interconnections Between BPD and ADHD

A meta-analysis performed in 2016 demonstrated that while most studies found deficits in some cognitive domains in individuals with BPD, no cognitive domain was consistently found to be impaired [63]. However, comorbid conditions, which are common in groups with BPD, need to be taken into consideration. Importantly, ADHD is rarely noted as a comorbidity in these instances [25]. Nevertheless, studies show that there is considerable overlap between the two conditions: about 14% of people diagnosed with ADHD as children go on to receive a diagnosis of BPD, and an estimated 18% to 34% of adults with ADHD are found to have comorbid BPD [64]. In adults, a childhood diagnosis of ADHD is a strong predictor of an increased risk of BPD, chiefly because of their common impulsivity [65]. Neglecting to take ADHD comorbidity into consideration in BPD research may lead to biased findings in cognitive performance [25]. Studies indicate that patients with BPD with comorbid ADHD exhibit greater cognitive impairments compared to both healthy controls and patients with BPD without ADHD [25]. Notably, working memory deficits have been observed in samples of patients with BPD where ADHD comorbidity was excluded [25]. Research by Linhartová highlights that patients with BPD without comorbid ADHD report increased impulsive choices and self-reported impulsivity but display intact impulsive action and cognitive functioning [25].

From a neurobiological perspective, impulsivity associated with BPD and ADHD involves distinct brain regions [66]. BPD-related impulsivity is linked to abnormalities in the prefrontal cortex and limbic system, whereas impulsivity in ADHD is associated with structural changes in the caudate nucleus and frontal–striatal pathways [66]. These findings suggest that despite overlapping behavioral symptoms, the underlying neurobiological mechanisms of impulsivity differ between the two disorders [66]. In general, ADHD and BPD share some common characteristics but also exhibit significant differences [64,66,67], which are outlined in Table 1.

### 4.3. Targeting Impulsivity in Borderline Personality Disorder

A wide variety of interventions have been explored for treating impulsivity in borderline personality disorder. The main approach that has received attention in research is psychotherapy, especially the forms that have been shown to be effective in treating impulsivity [29]. Psychotherapeutic treatment methods like dialectical behavior therapy (DBT), schema therapy (ST), and mentalization-based therapy (MBT) have shown significant promise in improving impulsivity and emotional regulation in BPD [29]. Additionally, the Systems Training for Emotional Predictability and Problem Solving (STEPPS) program has been identified as a valuable adjunctive treatment for BPD [68]. By providing a systematic, skills-based framework, STEPPS promotes emotional regulation and problem-solving skills, thereby contributing to reductions in impulsive behaviors [68].

In addition, pharmacological approaches to impulsiveness in BPD have been considered, although there are currently no drugs that have specifically gained approval for BPD treatment [69]. Different pharmacological treatments have produced inconsistent and inconclusive results [68]. Lithium has shown efficacy in targeting impulsivity in BPD in sporadic studies, despite small sample sizes and methodological issues, but its potential role remains to be clarified [70]. There is also some evidence on the use of lamotrigine for impulsivity [71]. Moreover, aripiprazole may reduce impulsivity and interpersonal problems [72], while Quetiapine may relieve affective instability and aggression [73]. However, the evidence is still in question, and patients with BPD may encounter considerable side effects. Thus, further randomized controlled trials (RCTs) must be performed to affirm the results [29,69].

Mood stabilizers, including lamotrigine and topiramate, have also been evaluated for their impact on impulsivity in BPD. Lamotrigine appears to show promise regarding treating some of the core symptoms of BPD, especially impulsivity and emotional dysregulation, but further RCTs are warranted [71].

Emerging neuromodulation techniques, such as repetitive transcranial magnetic stimulation (rTMS), offer additional avenues for addressing impulsivity in BPD [74]. Preliminary studies suggest that rTMS may modulate neural circuits involved in impulse control, warranting further exploration in this area [74].

## 5. Methylphenidate and Borderline Personality Disorder

Studies on the use of methylphenidate (MPH) in patients with BPD are limited and predominantly involve individuals with comorbid ADHD.

A study focused on patients with comorbid BPD and ADHD being treated with DBT, with some patients also receiving MPH or dexmethylphenidate treatment [75]. At baseline, patients with comorbid BPD-ADHD showed significantly higher levels of impulsivity than patients with BPD alone [75]. There were also significant improvements in anger symptoms, impulsivity, the severity of depressive symptoms, and ADHD severity in patients taking MPH as opposed to those not on stimulant medications [75].

Another study conducted in 14 adolescent patients with BPD-ADHD comorbidity treated with MPH demonstrated a global improvement in symptoms attributed to both ADHD and BPD, with an excellent tolerability profile [76]. Notably, core BPD symptoms such as impulsivity and aggression improved significantly, with some individuals achieving the remission of self-injurious behaviors [76].

A separate investigation on 22 patients with BPD treated with a single dose of MPH evaluated its impact on cognitive functions, particularly decision-making. Results indicated significant improvements in decision-making among those treated with MPH [77]. This is especially relevant as impaired decision-making in patients with BPD has been linked to greater impulsivity and more severe borderline symptomatology [78]. Additionally, lower inattention scores were associated with greater improvements in decision-making following MPH administration compared to higher ADHD symptom scores [77].

Observations made in case reports have described ameliorated symptoms in subjects with BPD-ADHD comorbidity following MPH treatment [79,80].

In addition, a real-life study conducted through the Swedish nationwide register databases showed that pharmacological treatments aimed at increasing the levels of extracellular dopamine and norepinephrine, such as bupropion, lisdexamfetamine, and MPH, were associated with enhanced outcomes in patients with BPD in real life. Although this class of medication is primarily used for ADHD, its efficacy in patients with ADHD symptoms from the BPD population may explain these results [81]. Additionally, an analysis of 22,601 individuals with BPD from the same registry found that ADHD medications, including MPH, were the only pharmacological option associated with a statistically significant reduction in the risk of suicidal behavior [82]. Given that impulsivity is a key predictor of suicidal behavior in BPD [59,60,61], these findings underscore the potential of MPH and similar medications in mitigating such risks. To understand why MPH might work for impulsivity in BPD, we should also briefly consider the neurobiology of BPD and the impact of MPH on different cerebral regions. In BPD, the dysregulation of prefrontal–limbic circuits, including the prefrontal cortex (PFC), amygdala, anterior cingulate cortex (ACC), orbitofrontal cortex (OFC), and striatum, has been observed [7,83]. These regions play a crucial role in impulse control and emotional regulation [7,83]. MPH enhances catecholaminergic transmission in the PFC, thereby improving attention, working memory, and impulse regulation [84]. Moreover, studies in ADHD have demonstrated that MPH also increases the activity of the striatum, leading to improved impulse control [85]. Although direct evidence in BPD is lacking, MPH may exert similar effects in individuals with BPD.

Therapeutic doses of methylphenidate are generally considered safe; however, the misuse or abuse of this medication can worsen psychiatric symptoms ranging from anxiety to depression and mood disorders [86]. It is worryingly reinforcing, thus posing a risk of addiction [87]. Methylphenidate misuse itself may produce a variety of changes in the human body [38]. These effects may include reduced appetite, gastrointestinal disturbances, headache, dizziness, increased sweating, and adverse cardiovascular effects such as tachycardia and variations in blood pressure [38]. Sleep disorders such as insomnia and daytime sleepiness are also common in these cases [38].

One might express concern regarding the prescription of methylphenidate for those suffering from BPD due to their highly impulsive natures [59]. The potential for abuse and dependency is thus even more disturbing since these can aggravate existing psychiatric symptomatology and complicate the management of BPD [87]. Accordingly, the evaluation and monitoring of any such treatment with methylphenidate should be conducted even more carefully in such patients [87].

It is also noteworthy that patients with BPD often present with other psychiatric comorbidities, such as anxiety disorders, depression, bipolar disorder, or SUDs [88]. Thus, when considering MPH as a treatment for impulsivity in BPD, a comprehensive assessment of comorbid conditions must be conducted. For instance, MPH might exacerbate anxiety problems [86]. MPH could also pose a risk of abuse in individuals with SUDs, although preliminary studies highlight its controlled use as a potential treatment for SUDs themselves [37].

## 6. Discussion

Impulsivity is a core feature of BPD, both for its impact on the overall symptom profile and for its association with the disorder’s prognosis [52,53,54]. This domain is also shared by other conditions, such as SUDs, BD, and ADHD [4]. In particular, BPD and ADHD display notable overlap, given their shared symptoms and relatively frequent comorbidity [25]. Although various psychotherapeutic approaches have proven effective in treating BPD and some of its core features, clear evidence regarding pharmacological treatments remains lacking [29]. To date, MPH has scarcely been investigated in BPD, despite its well-established efficacy for ADHD-related domains—such as impulsivity—that also manifest in BPD [49]. Indeed, a few studies have demonstrated the effectiveness of this medication for impulsivity in patients meeting diagnostic criteria for both disorders [66,67].

BPD has a complex etiology grounded, according to the biopsychosocial model, in multiple domains—including family relationships, traumatic events, and abuse experiences [89]. However, several studies over time have highlighted neurobiological alterations, particularly involving neurotransmitter systems such as serotonin and dopamine [83]. Alterations in dopamine, for instance, have been proposed to influence several BPD domains, including emotional dysregulation, impulsivity, and cognitive–perceptual impairment [90].

As described in the Mechanism of Action Section, MPH interacts with the dopaminergic neurotransmitter system by inhibiting the DAT [37,38,39].

In populations without BPD, the relationships among MPH treatment, the dopaminergic system, and impulsivity have been explored. One study in individuals with ADHD provided preliminary evidence that dopamine transporter occupancy by MPH in the putamen leads to improvements in cognitive impulsivity in adult male patients with ADHD [91]. Specifically, higher occupancy of dopamine transporters by MPH was associated with better cognitive impulsivity [91]. Another study in healthy participants correlated impulsivity, measured by the BOS, with DAT availability in the striatum [92], a region known to modulate dopamine release and regulate impulsive behaviors, as also shown in previous research [93]. In particular, higher DAT availability was linked to increased impulsivity [92]. Furthermore, a study involving adolescents with ADHD treated with MPH found that the medication increased dopamine neurotransmission in the striatum [94]. Remarkably, this augmentation of dopaminergic activity was associated with inattention and impulsivity severity, showing that the therapeutic benefits of MPH seem highly dependent on its striatum dopamine modulation properties—again, specifically in patients who have more severe symptoms of ADHD [94].

In patients with ADHD, studies have demonstrated a correlation among MPH use, its impact on dopaminergic dysfunction, and impulsivity [77,80]. This association could also extend to the treatment of patients with both ADHD and BPD, although further research employing similar methodologies in populations with these diagnoses is required to confirm this possibility.

## 7. Limitations and Conclusions

The main limitation of our review is the paucity of clinical studies on the use of MPH in patients with BPD. Although some studies have explored MPH treatment in patients with ADHD with comorbid BPD, more research specifically focused on patients with BPD is needed to reach conclusive results. This review also aims to highlight an unresolved issue: the unmet need for effective treatment targeting one of the most concerning symptom dimensions of BPD—impulsivity. In this regard, while diagnostic categorization remains essential, in clinical practice, addressing the core symptom—impulsivity—may be even more valuable, regardless of the specific diagnosis. Moreover, in naturalistic clinical settings, it is often encountered that BPD is misdiagnosed as ADHD, given their frequent comorbidity [25,67]. In these patients, MPH may play a role in managing their impulsivity.

However, large-scale studies, particularly including persons with a primary BPD diagnosis, are warranted to confirm the treatment’s efficacy in this population.

## Figures and Tables

**Table 1:** Commonalities and differences between borderline personality disorder (BPD) and attention-deficit/hyperactivity disorder (ADHD).

Domain	Borderline Personality Disorder (BPD)	Attention-Deficit/Hyperactivity Disorder (ADHD)
Impulsivity	Core feature, linked to risky behaviors (e.g., substance use, self-harm, suicidality). Impulsivity often triggered by intense emotions.	Core feature, primarily cognitive and motor impulsivity, difficulties with response inhibition and self-regulation.
Comorbidity	Often comorbid with bipolar disorder, substance use disorders, and eating disorders.	Higher rates of impulsivity-related behaviors. Can be comorbid with substance use disorders
Pharmacological Treatments	No approved treatments.	Stimulants (e.g., methylphenidate, amphetamines), non-stimulants (e.g., atomoxetine).
Response to Methylphenidate	Limited evidence, may reduce impulsivity in BPD with ADHD comorbidity, but studies on primary BPD lacking.	Well-documented efficacy in reducing impulsivity and improving cognitive control.

## Data Availability

Data are contained within the article.

## Abbreviations

The following abbreviations are used in this manuscript:

5-CSRTT	5-Choice Serial Reaction Time Task.
ACC	Anterior Cingulate Cortex
ADHD	Attention-Deficit/Hyperactivity Disorder
BD	Bipolar Disorder
BIS	Barratt Impulsiveness Scale
BOS	Behavioral Observation Scale
BPD	Borderline Personality Disorder
CBT	Cognitive–Behavioral Therapy
CM	Contingency Management
DAT	Dopamine Transporter
DBT	Dialectical Behavior Therapy
DSM-5	Diagnostic and Statistical Manual of Mental Disorders, Fifth Edition
DSM-5-TR	Diagnostic and Statistical Manual of Mental Disorders, Fifth Edition Text Revision
fMRI	Functional Magnetic Resonance Imaging
HiTOP	Hierarchical Taxonomy of Psychopathology
MBT	Mentalization-Based Therapy
MPH	Methylphenidate
NE	Norepinephrine
NET	Norepinephrine Transporter
OFC	Orbitofrontal Cortex
PET	Positron Emission Tomography
PFC	Prefrontal Cortex
RDoC	Research Domain Criteria
RCT	Randomized Controlled Trial
SST	Stop-Signal Task
STEPPS	Systems Training for Emotional Predictability and Problem Solving
SUD	Substance Use Disorder
VMAT2	Vesicular Monoamine Transporter 2
